# Antibiotic Economies: The Economisation of Antibiotic Use in Australia and Implications for the Mitigation of Antimicrobial Resistance

**DOI:** 10.1111/1467-9566.70011

**Published:** 2025-02-04

**Authors:** M. Davis, A. Schermuly, A. Rajkhowa, K. Thursky, N. Warren, P. Flowers

**Affiliations:** ^1^ School of Social Sciences and Centre to Impact AMR Monash University Clayton Australia; ^2^ Department of Infectious Diseases National Centre for Antimicrobial Stewardship Melbourne Medical School University of Melbourne Parkville Australia; ^3^ Department of Psychology University of Strathclyde Glasgow UK

## Abstract

This paper examines how economic rationalities shape antibiotic usage with the aim of expanding options for the reduction of antimicrobial resistance (AMR). Antibiotic usage is typically attributed to the individual behaviours of patients, pet owners and prescribers, an emphasis that has neglected sociological explanations, particularly the economic rationalities that are transforming healthcare. We used sociological theory of pharmaceutical capitalisation and economisation to explore in‐depth interviews on antibiotic usage with scientists, policymakers, prescribers, patients and pet owners in Australia. Antibiotics attracted values in terms of cost to the patient and pet owner, profit for the clinic, how the drugs saved time away from work and childcare, and how they eased the pressures of self‐care, parenting and pet ownership. Economic transactions that are only partially under individual patient and prescriber control shape antibiotic use. In these circumstances, antibiotic use is influenced by other social agents—for example, business managers and clinic owners—decentring prescriber authority. Adoption of socio‐economic values of antibiotic usage and inclusion of its other economic agents is needed to improve AMR intervention effectiveness.

## 1 Introduction

This paper examines the economic relations of antimicrobial use in Australia to inform public policy on antimicrobial resistance (AMR). Public health efforts on AMR generally focus on the large‐scale drivers of antimicrobial use, including health systems, regulation, clinical guidance, surveillance, intersectoral cooperation, public awareness and conducive economic settings. When policy and research does focus on actual antimicrobial usage, the individual knowledge, skills, predispositions and motivations of prescribers and users predominate. In what follows, we explore antimicrobial usage as constituted in relations that are shaped by culturally variable and shifting economic rationalities, including the local business models of healthcare provision and related practices of self that are centred on responses to infections and their impacts in everyday life.

Deeper understanding of the economic systems of antimicrobial usage is vital for optimising public policy efforts, particularly under conditions of the economic transformation of health systems. Microbes quickly evolve resistance to the antimicrobials used to control them. Unfortunately, resistance can make some infections difficult to treat and life threatening. In addition to significant mortality and morbidity (Antimicrobial Resistance Collaborators 2022), the global cost of AMR is estimated to be $100 trillion USD by 2050 (UK Review on Antimicrobial Resistance 2016). Considerable global effort is now underway to mount responses to the AMR threat. Global (World Health Organization 2015) and national (Australian Government 2021; HM Government 2019) action plans have been developed to coordinate action. Despite these efforts global surveillance of antimicrobial use shows that total consumption has continued to grow. Although some nations have shown reduction in antimicrobial use in human health (Van Driel et al. 2022) and animal agriculture (Wallinga et al. 2022), global antimicrobial use continues to expand (Roberts and Zembower 2021), including in low‐ and middle‐income countries (LMICs) undergoing rapid economic development (Klein et al. 2021; Van Boeckel et al. 2014, 2015).

It is also important to recognise that the global picture of antimicrobial use, AMR and their economic drivers tends to mask the quotidian materiality of getting drugs into the bodies of humans and animals. Obtaining antimicrobials via primary care, vet clinics, online stores, family members, or in local markets, to name a few examples, can all be construed as economic practices (A. Tompson et al. 2021) since each is shaped by social relations that have financial elements and are shaped by economic assumptions and meanings. Antimicrobial use can be construed as part of the socioeconomic infrastructures of “lives and livelihoods” (A. Tompson and Chandler 2021, 17). Antimicrobials, by reducing the impact of disease, enable economic activity for individuals and families, employers and the economy in general. This way of framing antimicrobials draws attention to the macro‐ and micro‐economic relations that are necessary for antimicrobial use to have material effects in life worlds.

In this paper, therefore, we consider why antimicrobial use has proven intractable in light of economic factors. Our approach follows calls for the increased application of theory‐informed qualitative research on AMR to provide much‐needed critical insights (van den, Bergh, and Brink 2021). We have adopted a One Health approach (Rubin, Baekkeskov, and Munkholm 2023), which seeks to promote collaboration and synergies across human and animal health, agriculture and the environment. Our approach contributes much‐needed sociological perspectives to the predominantly clinically‐focussed and clinician‐led social research on AMR (Charani and Holmes 2019; McKenna and Gale 2022). In the next section, we outline how the corporate imperative of pharmaceutical capitalisation undermines efforts to control AMR, imbuing the field with a significant paradoxical quality. Against this backdrop, we then draw attention to the values—economic and otherwise—attributed to the usage of antimicrobials. Our aim is to develop sociological insights regarding the production of AMR so that it can be more effectively controlled and hopefully reduced.

## 2 Background

### 2.1 The Economic Drivers of AMR: Power and Paradox

It is recognised that antimicrobial use is shaped by economic factors. For example, because antimicrobials are seen to be no longer profitable (UK Review on Antimicrobial Resistance 2016), pharmaceutical corporations are disinclined to invest in the discovery and production of new antimicrobial treatments to replace the ones that are beginning to fail. For example, if a company did try and sell a new patented antibiotic it would have to compete with existing lapsed patent antibiotics, and therefore the company would not see much profit. In addition, the time‐period between discovery and use of a new antibiotic can be up to 15 years due to the complexity of clinical trial methods and the drug approval systems that exist in different jurisdictions. In response to these challenges, it has been proposed that companies should be provided with cash incentives to invest in antibiotic research: as much as $1 billion USD for a new drug (UK Review on Antimicrobial Resistance, 2016). *CARB‐X*, *Combating Antibiotic‐Resistant Bacteria*, is a consortium funded by governments and the Bill & Melinda Gates Foundation to support research for new treatments (Årdal et al. 2020). Analysts have also recommended a single global system of drug approval and modifying effectiveness criteria in clinical trial methods to reduce costs for corporations (Hall, McDonnell, and O’Neill 2018; UK Review on Antimicrobial Resistance 2016).

However, there is a paradox at the heart of antimicrobial economics (Peterie et al. 2023). Containing antimicrobial usage to reduce AMR conflicts with corporate profit rationality, that is, the expansion of usage to increase profits (Roope et al. 2019; UK Review on Antimicrobial Resistance 2016). Pharmaceutical corporations invest in drugs and diseases that are most likely to yield results for shareholders, such as painkillers and Viagra (Dumit 2012). AMR, then, is troublingly enmeshed with pharmaceutical capitalism: the profit imperative requires increased use of antimicrobials, thereby contributing to AMR; the reduction of antimicrobial usage to control AMR is at odds with how corporations function to generate profit. In an attempt to address this paradox, it has been recommended that the link between sales and profit be severed to “eliminate incentives to oversell antimicrobials” (Glover et al. 2023, 1). One mechanism tried in the NHS has been to fund the acquisition of antimicrobials for a fixed price of £200 million pounds over a 10 year period (Glover et al. 2023). This method was thought to protect pharmaceutical companies from loss of income due to AMR policy that encouraged limits on the use of antimicrobials.

However, manipulating economic incentives for antimicrobial use does not fully address the core economic paradox of efforts to address AMR. Specifically, they support the income accumulation logic of pharmaceutical corporations and use public resources to do so. These public investments support the economic rationality that divides the value of corporate interests from the value of life‐saving antimicrobials. This difference between corporate and life‐saving values is explicit in increased incentives for private enterprise, as discussed above. Moreover, it is argued that the incentive value of a treatment option should be calculated in terms of its benefit for society—low toxicity, reduced resistance, treatment efficacy—so that a more socially valuable new treatment should attract more public support in the form of corporate compensation (UK Review on Antimicrobial Resistance, 2016). Although potentially helpful, in this reasoning it is accepted that corporate interests devalue life‐saving antimicrobials due to lack of profit, whereas they have value for society and must be supported by public investment. Under neoliberalised policy regarding healthcare, it has been assumed by some that the ‘market’ will naturally provide improved and efficient goods and services (Collyer 2015). The paradoxical effects of AMR economics are surely reason to doubt ‘market’ solutions.

Paradoxical AMR economics are echoed in other health domains. Immune modulation drugs used to reduce the rejection of transplanted organs have saved lives, but are also implicated in the development of organ markets and exploitation of majority world poor who sell their organs (Moniruzzaman 2019). The roll‐out of life saving HIV treatments was limited by the commercial interests of drug companies who claimed intellectual property rights and imposed costs that prohibited access for people with HIV in LMIC nations (Gostin and Rai 2020). Public and philanthropic resources were required to make life saving treatments available and commence the long struggle to eradicate the virus. These examples indicate that pharmaceutical capitalism is in itself paradoxically aligned with the betterment of health (Fox 2024). To borrow a phrase, pharmaceutical capitalism “contains within it the seeds of its own destruction, its own ‘grave diggers’” (Collyer 2015, 44).

### 2.2 Antimicrobials and Economisation

The counter‐productive effects of pharmaceutical capitalisation are significant, but they are not a total explanation for the drivers of AMR. Economic dynamics gain force and nuance due to their embeddedness in social systems (Swedberg and Granovetter 2011). For example, antimicrobials were not always of little value to corporations. In the post‐World War Two years, companies that included Lederle, Parke‐Davis and Pfizer expanded their fortunes through the marketing and sale of broad‐spectrum antibiotics (Podolsky 2015). The current low value of antimicrobials is an important example of the social and historical situatedness of what is considered valuable and valueless.

Relatedly, social practices are subject to “regimes of valuation” (Livne 2021, 896), that ‘economise’ by assigning and ordering values. Narrow economic values are joined by the moral, social and cultural valuing of objects, services, goods and people. In this view, antimicrobial use is not simply subject to cost and profit but ‘economised’ (Livne 2021), that is, antimicrobials are valued in terms of cost, but also for what they can do to treat infections and save lives, how they reinforce the power and status of medicine, and the roles they play in sustaining working lives, schooling and relatively harmonious parenting, to name some significant values.

Economisation also has significant implications for identity and relationality (Livne 2021). For example, Timmermans (2020) has documented the transformation of healthcare in concert with the rise of the neoliberal, engaged patient through the example of antimicrobials. He counterposed two audio‐recorded doctor–patient conversations—one from the 1970s in the UK and another decades later in California—to explore changes in the prescribing authority of the doctor and the transition to the engaged patient. The paternalistic instruction of the 1970s patient had given way to a patient‐led dialogue on symptoms and the eventual, but likely unwarranted, antimicrobial prescription. Timmermans argued that the engaged Californian patient is not simply a neoliberalised consumer making choices in a marketplace. Rather, they are caught between a supposed market for healthcare options and reliance on the expert knowledge possessed by the medical practitioner. This expertise has been decentred by managerialism, changed legal liability and new ethical considerations in risk management (Timmermans 2020), but it is not altogether sidelined. For this reason, Timmermans cautioned that the patient is not an outright consumer of healthcare in many national contexts, although some policy and marketing might assume them to be. More complexly, the prescribing relation is shaped by engaged patient—prescriber relations intersected by powerful and transforming economic dynamics. This perspective also depicts medical authority to be culturally‐specific through the example of the Californian doctor–patient relation. Timmermans's analysis also helps to focus on the clinical encounter, providing a rich mode of sociological inquiry on antimicrobial use in relational context.

The engaged patient and the part they have come to play in the prescribing relation can be construed as a reflection of the economisation of antimicrobial use under conditions of neoliberalism (Collyer 2015). Individuals find values in antimicrobials that fit with how they see themselves as responsibly engaged with their healthcare. This is a very different valuing of antimicrobials than that of pharmaceutical capitalisation where the drugs are not a source of profit. The values placed on antimicrobials by engaged patients are not directly calculated by corporations, but they do depend on these values to ensure that the public purse will continue to compensate them for their financial losses due to lack and loss of profit. This system of negative and positive antimicrobial values joins pharmaceutical capitalisation, the engaged patient and efforts to reduce AMR in ways that are both mutually supporting and antagonistic. It is, then, no wonder that the use of antimicrobials continues to grow and efforts to reduce AMR have not had much success.

In this paper, therefore, we seek critical insights into antimicrobial use in the Australian economic and regulatory setting and with respect to both human and animal healthcare. Through the sociology of pharmaceutical capitalisation and economisation, and in‐depth interviews with antimicrobial users, prescribers, scientists and policymakers we aim to understand how antimicrobial usage is valued—in the inclusive sense—in Australian settings.

## 3 Methods

To generate qualitative materials for our research we conducted 118 in‐depth interviews. Our interviews were comprised of 51 experts (scientists, policymakers and clinicians), 20 primary care doctors, 17 companion animal veterinarians and 30 members of the general public (14 with experience as pet owners). We adopted the principle of maximum variation purposive sampling to build our participant groups. Experts were selected according to balance of men and women, spread of roles in human, animal and environmental health, science and policy fields in Australia and the United Kingdom. We included UK experts to help reveal themes that were shared and not shared between the two nations and therefore to help build our theory of the economic drivers of AMR. Expert interviews focussed on experiences and views on AMR, One Health, policy and collaboration. Using a similar method, GP and veterinarian prescribers were recruited for a balance of gender, inner and outer urban clinic sites in Melbourne and mix of clinic types (small and big business, family and sexual health). Prescriber interviews focused on diagnosis and treatment of infections, AMR knowledge and strategies. Members of the general public were selected to provide an equal number of men and women, a range of ages from 18 to 70+, inner and outer urban place of residence, mix of parenting and pet ownership and self‐reported antimicrobial use in the previous 6 months. General public interviews generated experiential narrative on healthcare, use of antimicrobials and knowledge of AMR. 7/30 of the general public interviewees disclosed that they were men, 1/30 identified as non‐binary and 22/30 identified as women. The interviewees ranged in age from 20 to 77 years and all possessed a post‐secondary school qualification.

Interviews were conducted between March 2021 and June 2023 via Zoom due to COVID‐19 restrictions in Australia. Interviews were between 30 and 90 min in duration. All these were audio recorded and transcribed verbatim for input into NVIVO software. Interviewees provided electronic consent and audio‐recordings were transcribed verbatim and anonymised for analysis according to ethics clearance from the Monash Human Research Ethics Committee [number 26092].

The transcripts were analysed with a combination of thematic and narrative analysis (Squire et al. 2014). We first coded all materials using deductive themes identified from the research literature (Bryant and Charmaz 2019). Inductive themes that emerged through a method of constant comparison were also applied to materials. We generated coding reports and memoranda to identify and digest emerging themes. A series of ‘data labs’ were conducted where the research team collaborated to generate interpretations of key themes arising from the analysis, including the economics themes developed in this paper. During this analysis, we noticed that many informants used story‐telling methods to communicate about antimicrobial use, for example, representing a prescribing situation and its key social actors and using reported speech to depict the clinical encounter either as re‐creations or as notional imagined ones. The strongly dramaturgical quality of these particular interview fragments attested to the importance of the prescribing moment and its relational character for reflections on the economics of antimicrobial use and AMR. We then re‐analysed the materials to develop a narrative‐oriented analysis of lived experience, economics and antimicrobial use. We focused on depictions of clinical encounters, identities named (patient, vet doctor, practice manager, other family member and pet), reported speech, the positioning of the narrator and the prescribing outcomes.

## 4 Findings

We present our analysis as three related themes. In the first we explore how prescribers and experts spoke about cost considerations and antimicrobials, to establish important features of the economisation of antimicrobial usage. In the following section, we consider how patients, pet‐owners and prescribers narrated their experiences of antimicrobial use and therefore the significance of the engaged patient/pet‐owner for antimicrobial economisation. In the last section, we examine how economisation is associated with the dis‐embedding of the authority of the prescriber and shapes the autonomy of the user, with reference to implications for AMR.

### 4.1 Healthcare Costs and Antimicrobial Prescribing

The Australian setting has specific characteristics that have relevance for the economics of antimicrobial use. In Australia, human and animal healthcare is a mixture of state‐ and user‐pays. The national health insurance scheme (Medicare) supports primary care, and in this setting some patients pay no fee (referred to as bulk‐billed) or pay the difference between the state contribution and the fee charged by the GP. Primary care is delivered by small and big business in Australia, including large corporations (de Moel‐Mandel and Sundararajan 2021). Our practitioner and general public informants had experience of all these contexts, with some GPs reporting, for example, that they worked sessions in multiple clinics—small and large—in different parts of Melbourne, on a weekly basis. Oral antimicrobials are most commonly prescribed by a doctor and, under the Pharmaceutical Benefits Scheme (PBS), the cost to the patient is significantly reduced. In veterinary care for small animals and on farms, the cost of antimicrobials is borne by the user. Compared with other affluent nations (Davis et al. 2014), Australia is ranked highly for quality of healthcare but high for out‐of‐pocket cost for the patient, alongside the United States where a significant proportion of the population is underserved. The UK and Sweden are ranked highest for access reflected in least for out‐of‐pocket expenses.

Veterinary and GP clinics can also take on specific characteristics if they focus on particular groups, for example, veterinary clinics for cats or birds, GP clinics for family medicine, university students and staff, refugee health and LGBTIQ + health. This diversity is one reason for the high degree of health care users' reflexivity in the interview fragments to follow. Patients, in particular, appear to value being able to make choices regarding the form of care they wish to obtain, a feature supported by the underpinning philosophy of the Australian system.

Unsurprisingly, given the emphasis placed on choice, antimicrobials held nuanced economic meanings. Our interviewees noted that antimicrobials had varying relationships with the direct costs of healthcare and the indirect costs of illness. They also made reference to the time and money saving properties of antimicrobials. Fiona, who provided family medicine at a private clinic, spoke of antimicrobial prescribing in relation to the indirect costs for her patients:

… if they’ve come in with a sick kid and it’s a fairly undifferentiated problem, I say, “Look, we need to see them again in 24, 48 hours,” and we would mostly bulk‐bill that consultation … … because I think that really is a barrier for our patients. The other reason is sometimes, you know, the parents are under pressure to be at work, to get the kid back to childcare … … So those sorts of things are hard. So, I think financial pressures is one, time pressures is another …

(GP 03)

In the interview fragment, Fiona used the narrative method of reported speech to dramatise a conversation between herself and a parent seeking help with their child. This situation is represented as infused with the time and money imperatives that come with parenting and work. In the fragment, bulk‐billing refers to medical consultations that attract no fees, as described above. It reveals that antimicrobials are economised in terms of benefits for parents and not simply as valuable for the reduction of infections. They also have pronounced values for individual participation in the economy, that is, “lives and livelihoods”, as A. Tompson and Chandler (2021) have asserted. Fiona's example demonstrates how narrow economic considerations are embedded in social practices and contexts (Livne 2021).

Veterinarians also reported that costs shaped the care they were able to provide. Helen was a veterinarian working in inner Melbourne. In this example, she spoke of negotiating care and how outcomes for the pet were tied to the ability of the pet owner to pay:

Cost is definitely a big one. You know, animal medical care is really expensive and they don’t have Medicare. To give you an idea, a consultation itself is $300 and that’s before any treatment and medications. And the medications do add up ‘cause there’s a dispensing fee. Overnight hospitalisation with diagnostics is easily two and a half, three, four grand … … if their dog or cat is really unwell, then we always try, “This is the gold standard if we want to do everything. But, let’s try and work within your budget. Do you have enough just to be admitted for IV antibiotics overnight?” for example, if needed, and go back home the next morning. But, if it is things like, you know, septic abdomen, something that’s really serious, then it might be that we recommend euthanasia instead.

(Vet 13)

The example shows that cost considerations are factored into the course of action, in this hypothetical case, in relation to a severe infection. Like Fiona, Helen used reported speech to summarise a negotiation with the pet owner regarding costs and treatment. Her account implies that life and death decisions grounded in cost considerations are brought to the fore in animal health care, reinforcing the link between antimicrobials, costs and values. It also implies that affluent pet owners have more choices about the welfare of their pets, another way in which economic factors drive the delivery of antimicrobials into bodies.

### 4.2 Engaged Patients and Pet Owners

General public and clinician interviewees also showed awareness of the varied business models of human and animal healthcare and a related investment in personal autonomy over health matters. Angela was a retiree living in outer Melbourne with a background in bioscience. In their interview, Angela commented on a visit for her dog's dental care that included costly antimicrobials:

… I knew what she was choosing and I knew it’d be expensive, but I didn’t know it’d be that expensive. And when did I find out about the cost of that? What happened is she said, “I will talk to her boss about what happened. Don’t pay now. I don’t think we should charge you for this.” And then I didn’t hear. And about a month later I got a phone call from the clinic to say that I owed them $120 for the antibiotics. And I think she just had perhaps forgotten to talk to the boss or the boss had said, “No. She must pay for them.” So, I just paid for it but that’s the first time I realised how expensive it was. So, that’s a bit tricky. I mean I was grateful but … … And luckily, I have that much money.

(General public 08)

As with Fiona, Angela's interview fragment embeds cost in the social relations of veterinary medical care. Importantly, Angela's reported and probably invented speech brings at least one other individual—the boss—into the prescribing decision and its aftermath. In this account, prescribing is fused with the financial management of the clinic. Antimicrobials are multiply economised because they are therapeutic for the pet owner and a source of income for the clinic.

General practice care—despite it being mostly state‐supported—was also understood to have prominent economic nuances. In this example, Glenda, who was in her 60s and residing in inner Melbourne, made note of a tiered approach to payments for care:

I have two GPs that I use regularly. One doesn’t bulk bill and the other one does. So, if I have something where I want some tender loving care, I go to the GP who charges. And, if I just have an ordinary, simple problem, I’ll go to the bulk‐bill GP … I’m a domestic violence survivor so, if I want some mental health support, I’ll go to the GP that I pay for …

(General public 17)

Glenda provided a sense of herself allocating resources in a strategic manner. She saw that bulk‐billed (no fee) consultations were appropriate for issues that had less value, whereas mental health care was valued differently, and Glenda chose to pay out of pocket for the difference between government support and the fee charged by the practitioner. Investing in mental health and securing the right kind of treatment was worth paying for whilst other matters were managed more perfunctorily. Her example indicates that some patients are able to strategically manage the cost of different forms of healthcare and their value for body and mind. Glenda's hierarchy of mental health over “ordinary” health deftly economises mental and other healthcare. This management of cost and value echoes the engaged patient Timmermans (2020), of neoliberal economic systems.

Prescribers showed some awareness of the complex choices made by patients and pet owners. Diane (GP 19) worked in an inner Melbourne private practice that provided family medicine on a mixed billing basis. She talked about how doctors and patients navigated different kinds of practices:

I mean I would probably say that, unfortunately, as a broad‐brush statement, patients will go to GPs that they know will prescribe antibiotics and those doctors are probably doctors that have high‐flow, high‐input patients, and they’re trying to see people in under six minutes to get quick medicine. And those doctors are probably already burnt‐out, and not listening to what’s being said to them … … I worked in a clinic and if I said no to them, they would just walk out, sit down and book in with another doctor. And they’d go to the other doctor and they’d get the antibiotic. And, despite the fact that it wasn’t relevant and it wasn’t appropriate, they got it. So, you can’t work against that … … ‘Cause I’ve worked in clinics where the doctors are working in silos … … And they were fast, quick doctors who would wanna get, you know, seven to eight, to 10 people in an hour through, quick …

(GP 19)

Diane provided a picture of patients who reflexively manage the options before them in ways that mirror the previous fragments from Angela and Glenda. Her narrative indicates that this particular business model of primary care enables doctor shopping, that is, the patient would not be able to act without the collusion of the clinic and another medical practitioner. The references to burnout suggest rather pointedly how some business models create the conditions for doctor shopping and therefore may interrupt the prevention of AMR. Notably the high throughput clinic figures here as a model that facilitates doctor shopping and possibly increased access to antimicrobials. To the extent that this is true and has impact on AMR, patient education and clinical decision‐making tools are unlikely to have much traction in the face of these powerful economic drivers of antimicrobial use.

Notable too is the contrast between Diana's depiction of patients and the previous interview fragments provided by Angela and Glenda. Diana sees reflexive choice‐making on the part of patients as circumventing her authority over the use of antimicrobials. Angela and Glenda see choices as expressions of autonomy that are necessary given the varied services made possible in healthcare business models. These different prescribing approaches suggest clashing regimes of the valuation of antimicrobials (Livne 2021). In this example from Eloise, an inner urban GP working in a private clinic, patients are depicted as focussed on solutions due to their economic circumstances:

…people are busy and, you know, can’t afford or, either money‐wise or time‐wise, can’t afford to be sick, and don’t want to be diagnosed with something that requires time, time to rest and time away from work. And time to not infect other people. I think that’s the other thing people really don’t like is knowing in this climate (COVID‐19 pandemic) that they’re infectious with something that’s gonna infect other people. And they really love having a solution. It’s not just antibiotics: it’s people who do that same reaction when they’re told that all their blood tests are normal because, you know, they’re tired because of something that I can’t give them a medication for. I think people love to be diagnosed with a quick fix.

(GP 13)

It is significant that prescribers and users appear to have different values regarding the use of antimicrobials. Patients and pet owners create pictures of themselves as exercising rational volition over their own health and that of children and pets. Prescribers draw on the ‘Dr shopping’ narrative to explain the behaviour of individuals they see in their practices. This separation is noteworthy because patients, pet owners, GPs and vets are all speaking of the same social phenomenon—antimicrobial use and healthcare more generally—but from different and somewhat conflicting vantage points. Implied, therefore, is that the ontology of the clinical encounter is not a shared one, which might help to explain why some patients and pet owners question expert advice and why these practices trouble prescribers. Moreover, this separation of vantage points is intersected by the varied economic models of care and the different values placed on the transactions possible in those circumstances. In this framing, the clinical encounter is less one of an expert offering guidance to a patient and more akin to the negotiation of different ways of valuing antimicrobials.

### 4.3 Transformation of Authority and Autonomy

Interviewees also had much to say about the changing business models of primary and veterinary care and implications for their interactions with professionals. As we have noted, in Australia, primary care is state‐supported but clinics vary in terms of size, served population and business model. The work of GPs is also somewhat fragmented by this variation. This system of healthcare is undergoing considerable transformation as the Australian government seeks to improve services and rationalise cost and as health care provision changes in response, particularly in terms of newer options such as telehealth consultations and online prescriptions. Vet clinics are wholly private businesses. Our interviewees expressed awareness of these factors in healthcare and how they might impinge on the use of antimicrobials. Their personal experience narratives demonstrated how macro‐ and micro‐economic factors intersected in real world antimicrobial use.

Eunice was a retiree in her early 60s residing in outer Melbourne. In this interview extract, she commented on the transformation of healthcare and the deepening emphasis on time, efficiency and income:

… a lot of doctors work on a timer now, you know. For instance, our GP, he had his own practice. There was three of them. Absolutely fantastic. He sold his practice to a big conglomerate. Now, and he’ll tell you, like a little buzzer thing goes off if you’ve been in there longer than eight minutes. He said, “You’re s’posed to have a minute to bring the person into the room. A minute to see them out. And eight‐minute consultation. So, you have an appointment every 10 minutes.” I mean he does break that sometimes. He said, “You know, some people are in and out in five minutes if they just want a repeat script or whatever, then you’ve got others that are genuine, that take that extra time.” But he said, “Under the business guidelines of the corporation, this is what they want. They want people in and out.” And I think that’s sad because people aren’t getting the opportunity perhaps to talk about the things that they’re wanting to talk about.

(General public 10)

This account echoes Diane's references to high throughput clinics. It also narrates the transformation of family medicine and the related economisation of time spent with each patient. The efficient production of increased value appears to shape the clinical encounter. Similar economic dynamics appeared in the experiential accounts of veterinarians. Graham worked in an inner urban vet clinic. They reflected on their experience of working in different kinds of clinics and an unspoken pressure to sell products:

It’s varied over the years depending on who I’ve been working for. When I’ve sort of been working for a family‐owned business, it’s not too bad. But often there’s a few little suggestions here and there that, you know, we should be selling more products. And antibiotics could be one way of just increasing a little bit of revenue here and there. But it’s not, you could argue that it’s not gonna do any harm to the pets … … But there’s a bit of pressure in that way. From a lot of the corporate clinics, they really look at figures and numbers. And, if you are only pulling in a certain amount of revenue per appointment, they want you to try and do a little bit better. And like most corporations it’s always just trying to get you to do a little bit better. And, so a bit more pressure on the dollars per appointment or per person that you’re seeing. And that can be quite hard on vets, unless they’ve got their confidence or they don’t care what their bosses say. So, that can be a bit hard sometimes.

(Vet 02)

Graham indicated that economic imperatives were never absent but did vary in intensity depending on the business context. It is recognised that veterinary care for companion animals is subject to costs that can lead to unnecessary use of antimicrobials (Hardefeldt et al. 2018; King et al. 2018). Vets report that diagnostic tests that may assist with prescribing antimicrobials appropriately can be too costly for their patients and that because some of their income is derived from the sale of antimicrobials, prescribing them may be more attractive (Hardefeldt et al. 2018; King et al. 2018). Graham suggested that the corporate veterinary clinic intensifies these dynamics. Similarly, Lorraine, who worked in a private, family medicine clinic in inner Melbourne, reflected on the impact of a shift away from bulk‐billing:

The practice manager recently joined the team and the practice had changed from being a bulk‐billing practice, where everyone from the hospital just went in and got seen and treated, into a place where patients had to pay. So, they lost a large amount of their patient population ‘cause people were not used to paying. They didn’t want to pay. So, the practice manager’s approach was to keep everyone super‐happy and give them what they want, whereas my approach is that I’m a doctor, I’m not a shop. I’m happy to assess you but I’m not happy to give you what you think you need. I’m happy to work out a plan with you. So, her approach was basically keeping patients and making sure that the patient population wouldn’t drop further. But I don’t think that’s a good reason to do medicine. That’s not according to the guidelines or actually not really helping the patient.

(GP 15)

Lorraine suggested that, due to a change in billing arrangements from bulk‐billing (no fee) to consultations that attracted fees, some patients ceased attending her clinic leading to an increased focus on the financial survival of the clinic. According to Lorraine, this change meant that she experienced pressure to satisfy patients in ways that conflicted with her clinical judgement. The account echoed previous fragments which suggest that patients respond to the costs of healthcare; in this instance, highlighting patients as willing to move clinics if the costs become prohibitive. But in contrast with the view that the high throughput clinic was associated with reduced quality care, in Lorraine's account, business imperatives in billing practice also interfered with the exercise of clinical judgement. The phrase “I'm not a shop” indicated some resentment towards these changed conditions on the part of the practitioner and a related diminution of authority over prescribing decisions. It signified a clash of values and therefore contradictory currents in the economisation of antimicrobial use. To the extent that acquisitive economic rationality casts the seeds of its own destruction (Collyer 2015), it seems possible to say that ‘marketised’ healthcare is consuming medical authority at pace, with implications for patient safety and the reduction of AMR.

Key here is that the action of the antimicrobial user depends on the collusion of the business managers. The patient or pet owner cannot easily act alone; the business setting facilitates their action. In this view, patient demand is a misnomer because it does not exist outside of a system organised in ways to make it possible. Moreover, the decision to prescribe is emptied of its medical authority and is primarily a transaction between the patient or pet‐owner and the business managers. The story reveals the forces outside of the clinician–patient relation that shape the encounter, forces that are said to trump the authority of the prescriber.

These extracts reinforce the view that business models shape clinical encounters and therefore antimicrobial use. They also suggest the transformation of the clinical encounter from one figured around the medical/veterinary expert and the compliant patient/pet‐owner to one that comprises a provider catering to an engaged patient/pet‐owner. In this transformation, economic factors to do with the running of the clinic appear to have prominence. This transformation reflects public policy shifts which seek to counter unhelpful medical paternalism and strengthen the rights of patients (Timmermans 2020). It is also consistent with increased emphasis on the engaged patient or pet‐owner, who is required to take personal responsibility for their care. But these shifts appear to also be linked with a shifting landscape of prescribing authority shaped by changing business models and especially the notional corporate clinic. In this context the time and money saving values of antimicrobials attain special status as attractive solutions. It could also be argued, by reversal, that time and money saving antimicrobials help to make the economic rationality of high throughput healthcare possible.

Together these fragments provide a richly nuanced picture of economisation in human and animal health care, its impact on prescribing and how patients and pet owners are seen to navigate these changes. The interview fragments also show that prescribing encounters are not simply a binary of expert and user. Other individuals play a role in the management of economic efficiencies and therefore prescribing decisions. This enmeshment of the clinical encounter with economic rationality links with other healthcare developments. Pharmacy prescribing and online scripts are other ways of delivering antimicrobials that depend on and celebrate a notional consumer (NSW Health 2023). These transformations have implications for the status of prescribing authority but have yet to be fully recognised in policy settings on AMR (Australian Government 2021).

## 5 Conclusion

Our analysis has developed an account of the economics of antimicrobial use to gain insight into the proliferation of AMR. Antimicrobials were thoroughly and multiply economised in terms of cost to the patient and pet owner, profit for the clinic, how the drugs saved time away from work and childcare and how they eased the pressures of self‐care, parenting and pet ownership. Access to antimicrobials was also influenced by the time allowed for consultations and profit‐making imperatives, which varied by the business model of the clinic. In particular, corporation‐style imperatives for profit and efficiency were understood to help shape prescribing. Antimicrobial use was infused with the identities of the engaged patient/pet owner and the expert prescriber, but not always harmoniously. Antimicrobial use provided a way for the engaged patient to secure their identity and perform responsible action for themselves and loved ones. Interviewees found value in making choices about their healthcare and created hierarchies of value to help guide those decisions. Expert prescribers found that their identities as guardians of patient and pet safety were put into question when patients asserted choices and ‘shopped around’. Moreover, clinic managers and other agents of economisation also contributed to the costing of antimicrobial use and, it seems, decisions to use them in the first place.

Consistent with the embeddedness of economic values, using antimicrobials is deeply relational, involving general practitioners, veterinarians and members of the general public, along with other social actors, including clinic managers and owners. For example, the organisation of the GP clinic manufactures antimicrobial use because it permits access to prescribers willing to consent to an unnecessary antimicrobial prescription amplified, perhaps, in a context of high‐throughput medicine. In the veterinary clinic, cost, time and business imperatives help to drive the unnecessary use of antimicrobials. Some prescribers resist these moments—as Lorraine (GP 15) did when she said “I am not a shop”—but also find themselves usurped by the business configuration of the clinic. The repeated use of dramaturgical reported speech when interviewees were explaining their experiences underlines the interpersonal transactions that make antimicrobial use possible. A high degree of reflexivity with this situation was apparent among prescribers and users, including with the complex and changing business models of human and animal healthcare.

Antimicrobial economisation plays out in ways that may limit the effective control of AMR. The authority of the prescriber is somewhat decentred by the alliance of profit‐oriented business models and the engaged patient. Patient and pet owner valuing of choice clashes with prescriber valuing of expert authority. Making choices about healthcare reflects neoliberalised economic rationality which is at odds with the reduction of unnecessary antimicrobial use and therefore the reduction of choice. Antimicrobial use has become a situation somewhat fraught by tensions that have their source in divergent regimes of valuation (Livne 2021). Improved efficiency and a focus on profit may have benefits for some forms of healthcare, in keeping with market‐oriented models of public policy, but not for antimicrobial use and AMR. This clashing of values mirrors the paradox of pharmaceutical capitalisation in which corporations seek expansion of markets and therefore see no value in antimicrobials, especially if antimicrobial use has to be contained and reduced. Further, healthcare business models have come to emulate pharmaceutical capitalism because they share a principle of income accumulation through increased efficiency. This imperative is another way in which prescribers are pressured and have their authority eroded. These economic factors are not easily controlled by the individual, even if they believe they are making choices about their healthcare. These choices are exercised under the conditions of possibility tied to the economisation of antimicrobials and through them healthcare.

Economised antimicrobial use indicates the need to revisit the economics of antimicrobial use in AMR policy. AMR policy often conceptualises antimicrobial use in community settings as a technocratic exercise shaped by the knowledge, skills and attitudes of prescribers and users (World Health Organization 2015). Our analysis draws attention to use enmeshed with the economic values that are seen to make the clinic viable. We found that unnecessary prescribing occurs despite the knowledge and skills of prescribers. The economisation of antimicrobial use drives prescribing in ways that exceed the typical individual behaviour focus of AMR interventions (Price et al. 2018). It follows, therefore, that policy settings need to address micro‐economic factors. According to the economisation point of view, shaping antimicrobial use will require the cooperation of other important actors—business managers, childcare centre managers and business owners—in order to be effective. Integrating AMR policy into clinic business models will require consultation with these other shapers of the economisation of antimicrobial usage. This economisation gaze on antimicrobial use is also relevant for countries subject to high levels of self‐medication and with poorly resourced public health infrastructures (Kalam et al. 2021). In those settings, consulting with informal marketers is likely to be vital.

AMR provides an important lens on the sociology of economy since antimicrobials have little value for corporate capitalism yet have life‐saving powers. This paradox brings an important new dimension to theory because it makes apparent the valueless status of life of species under some versions of capitalism (Collyer 2015). Efforts to preserve life—in this example, by rationalising use of antimicrobials to limit AMR—appears to conflict with corporate capitalism. This seems to be true for pharmaceutical corporations that need to be compensated for them to continue to produce antimicrobials and in clinics where business models are unfriendly to medical authority over the rationalisation of antimicrobial use. It is commonplace to critically reflect on corporate interest in high value goods and services and how those investments are expanded and protected. It is less common to consider how low value goods are also enmeshed in economic rationality. Antimicrobials are an important means of nuancing the sociology of economy to embrace objects that are paradoxically valueless and valuable. Indeed, life under advanced capitalism depends on this paradox.

## Author Contributions

**M. Davis:** conceptualization (lead); data curation (lead); formal analysis (lead); funding acquisition (lead); investigation (lead); methodology (lead); project administration (lead); resources (lead); supervision (lead); writing–original draft (lead); writing–review & editing (lead). **A. Schermuly:** data curation (supporting); formal analysis (supporting); project administration (supporting). **A. Rajkhowa:** data curation (supporting); formal analysis (supporting); project administration (supporting). **K. Thursky:** conceptualization (supporting); formal analysis (supporting); funding acquisition (supporting); investigation (supporting); writing–review & editing (supporting). **N. Warren:** conceptualization (supporting); formal analysis (supporting); funding acquisition (supporting); writing–original draft (supporting); writing–review & editing (supporting). **P. Flowers:** conceptualization (supporting); formal analysis (supporting); funding acquisition (supporting); investigation (supporting); writing–review & editing (supporting).

## Data Availability

The data that support the findings of this study are available on request from the corresponding author. The data are not publicly available due to privacy or ethical restrictions.
